# Predictive significance of systemic immune-inflammation index combined with prealbumin for postoperative pneumonia following lung resection surgery

**DOI:** 10.1186/s12890-024-03086-7

**Published:** 2024-06-11

**Authors:** Haihang Miao, Dingying Ge, Qianwen Wang, Lulu Zhou, Hongsheng Chen, Yibin Qin, Faqiang Zhang

**Affiliations:** 1 Department of Anesthesiology, Affiliated Hospital of Nantong University, Nantong, China; 2 Department of Oncology, The First Affiliated Hospital of Jinzhou Medical University, Jinzhou, Liaoning Province China; 3 Department of Anesthesiology, Shanghai Pulmonary Hospital, Tongji University School of Medicine, Shanghai, 200433 China

**Keywords:** Postoperative pneumonia, Prealbumin, Systemic immune-inflammation index (SII), Lung resection surgery

## Abstract

**Background:**

We aimed to determine whether systemic immune-inflammation index (SII) combined with prealbumin can provide better predictive power for postoperative pneumonia in patients undergoing lung resection surgery.

**Methods:**

We identified eligible patients undergoing lung resection surgery at the Affiliated Hospital of Nantong University from March 2021 to March 2022. Demographic characteristics, clinical data, and laboratory information were collected and reviewed from the electronic medical records of the patients. To test the effect of the combined detection of SII and prealbumin, we made an equation using logistic regression analysis. The receiver operating characteristic curve (ROC) was plotted to evaluate the predictive powers, sensitivity, and specificity of prealbumin, SII, and SII combined with prealbumin. Decision curve analysis (DCA) was used to determine the clinical validity and net benefit of different methods of detection.

**Results:**

Totally 386 eligible patients were included with a median age of 62.0 years (IQR: 55.0, 68.0), and 57 (14.8%) patients presented with postoperative pneumonia within 7 days after surgery. The multivariate regression analysis showed that preoperative SII as continuous variable was associated with an increased risk of postoperative pneumonia (OR: 1.38, 95% CI: 1.19–2.83, *P* = 0.011), whereas the prealbumin as continuous variable remained as an independent protective predictor of postoperative pneumonia in the adjusted analysis (OR: 0.80, 95% CI: 0.37–0.89, *P* = 0.023). Compared to SII or prealbumin, the combined detection of preoperative SII and prealbumin showed a higher predictive power with area under curve of 0.79 (95% CI: 0.71–0.86, *P* < 0.05 for all). Additionally, DCA indicated that the combined detection was superior over preoperative SII or prealbumin alone in clinical validity and net benefit.

**Conclusion:**

Both preoperative SII and prealbumin are independent influencing factors for postoperative pneumonia after lung resection surgery. The combined detection of preoperative SII and prealbumin can significantly improve prediction capability to identify potential postoperative pneumonia-susceptible patients, facilitating early interventions to improve postoperative quality of life for surgical lung resection patients.

**Supplementary Information:**

The online version contains supplementary material available at 10.1186/s12890-024-03086-7.

## Background

Postoperative pneumonia (POP) is a common complication after lung resection surgery and remains the main cause of postoperative morbidity and mortality [1]. Postoperative pneumonia, still occurs at a relatively high rate, despite measures such as lung protective ventilation strategy, antimicrobial therapy, and perioperative pulmonary rehabilitation training, which have proven effective in preventing postoperative pulmonary infections [2–4]. Currently, the search for simple, accurate tools, especially preoperative biomarkers, is a pressing challenge in clinical practice. These biomarkers may contribute to better identification of high-risk patients and timely implementation of targeted preventive measures.

Inflammation and malnutrition are ubiquitous in the perioperative period of patients undergoing elective surgery [5, 6]. The systemic immune-inflammation index (SII), a novel inflammation-related index, shows a favorable predictive value of postoperative pneumonia in surgical lung cancer patients [7]. However, this estimate of risks for postoperative pneumonia appears less stable. Another study of patients undergoing hip fracture surgery indicates that the SII remains limited in its predictive capacity for postoperative pneumonia [8]. Meanwhile, serum prealbumin is a relatively easily obtainable indicator, typically used alone or in combination with other biomarkers to reflect the nutritional status [9]. Preoperative low prealbumin or high C-reactive protein (CRP) to prealbumin can serve as a potential risk indicator for predicting the occurrence of postoperative pneumonia in patients undergoing spinal surgery or esophagectomy [10, 11]. Going further, inflammation and prealbumin are tightly linked and may affect each other [12]. To date, it is not known whether the addition of prealbumin to SII can further improve the predictive power or stability of the SII.

For better prediction of postoperative pneumonia, we have proposed an integrating detection of SII and prealbumin that organically reflects inflammatory and nutritional pathways. Herein, this study aimed to investigate the relationship between preoperative SII or prealbumin and the occurrence of postoperative pneumonia and determine whether SII combined with prealbumin can provide better predictive power for postoperative pneumonia in patients undergoing lung resection surgery.

## Materials and methods

### Study design

This study was reviewed and approved by the ethics committee of the Affiliated Hospital of Nantong University (No. 2022-K107-01). Due to its retrospective nature, the informed consent of the included participants was waived by the institutional review board. The study conformed to the Declaration of Helsinki and STROBE guidelines. All identifiable data were completely anonymized before analysis.

### Study population

We identified patients who underwent elective lung resection surgery at the Affiliated Hospital of Nantong University from March 2021 to March 2022. Eligible patients had to meet all the following criteria: (1) aged 18 years or older; (2) treated with elective lung resection surgery with video-assisted thoracoscope; (3) received general anesthesia with double-lumen endobronchial intubation and extubated in the operating room; (4) presented with an American Society of Anesthesiologists (ASA) classification I–III. We excluded participants with preoperative history of respiratory infection in the last 3 months, wedge resection of lung, and inability to calculate SII scores due to missing laboratory parameters. Additionally, clinical cases with a medical history of hepatic diseases affecting serum prealbumin levels were also excluded.

### Data collection and definition

Demographic characteristics, clinical data, and laboratory information were collected and reviewed from the electronic medical records of the patients by trained coordinators blinded to the study protocol. Preoperative baseline data, such as age, gender, body mass index (BMI), ASA physical status, smoking, drinking, hypertension, diabetes mellitus, coronary heart disease, and chronic obstructive pulmonary disease (COPD), were noted. BMI was calculated as weight (kg) divided by height squared (m^2^). Blood samples were obtained and processed within 3 days prior to surgery. Prealbumin, albumin, neutrophil, lymphocyte, platelet, hemoglobin, and total bilirubin were measured and documented. The preoperative SII was calculated based on the following formula: platelet count × neutrophil count/lymphocyte count. In addition, we collected surgical information, such as type of surgery, duration of surgery, blood loss, and total fluids.

### Clinical outcome

The primary endpoint of interest was the occurrence of POP in patients hospitalized with lung resection surgery within postoperative 7 days. POP was diagnosed during hospital stay according to a combination of neuroimaging and clinical analysis of chest X-ray examination (new or progressive pulmonary infiltrates), abnormal laboratory tests (leukopenia or leukocytosis), and the clinical symptoms or signs (cough, rales, fever, new onset of purulent sputum, or worsening gas exchange) [13]. Additionally, we only considered hospital-acquired pneumonia; we excluded pneumonia happened before surgery.

### Statistical analysis

Patient characteristics were expressed as numbers (percentage) for categorical data and median (interquartile range, IQR) for quantitative data. The Student’s t-test and Chi-square test were used to compare differences in characteristics between patients with and without postoperative pneumonia. To identify potential predictive factors of postoperative pneumonia and to evaluate the predictive significance, univariate and multivariate logistic regression analyses were performed. Variables associated with POP at a significance level of < 0.1 in univariate analysis or with clinical plausibility were subsequently entered into a multivariate logistic analysis using stepwise method. The SII, or prealbumin was converted into a two-level categorical variable based on the optimal cut-off point using the receiver operating characteristic curve (ROC). To test the effect of combined detection of preoperative SII and prealbumin, we made an equation using logistic regression analysis. Dose–response relationship between the combined detection and postoperative pneumonia was assessed using restricted cubic spline analysis, with three knots at the 25th, 75th percentiles, and the reference point at the median of combined detection. The area under the ROC curve (AUC) was determined to evaluate the predictive powers of different methods of detection. Decision curve analysis (DCA) was used to determine the clinical validity and net benefit. Variables with a *P* < 0.05 were considered statistically significant for all tests. All statistical analyses were conducted using SPSS Statistics 26.0 software (SPSS, IBM Corporation) and R version 4.0.5 statistical software (The R Foundation).

## Results

### Clinical characteristics

**Fig. 1:**
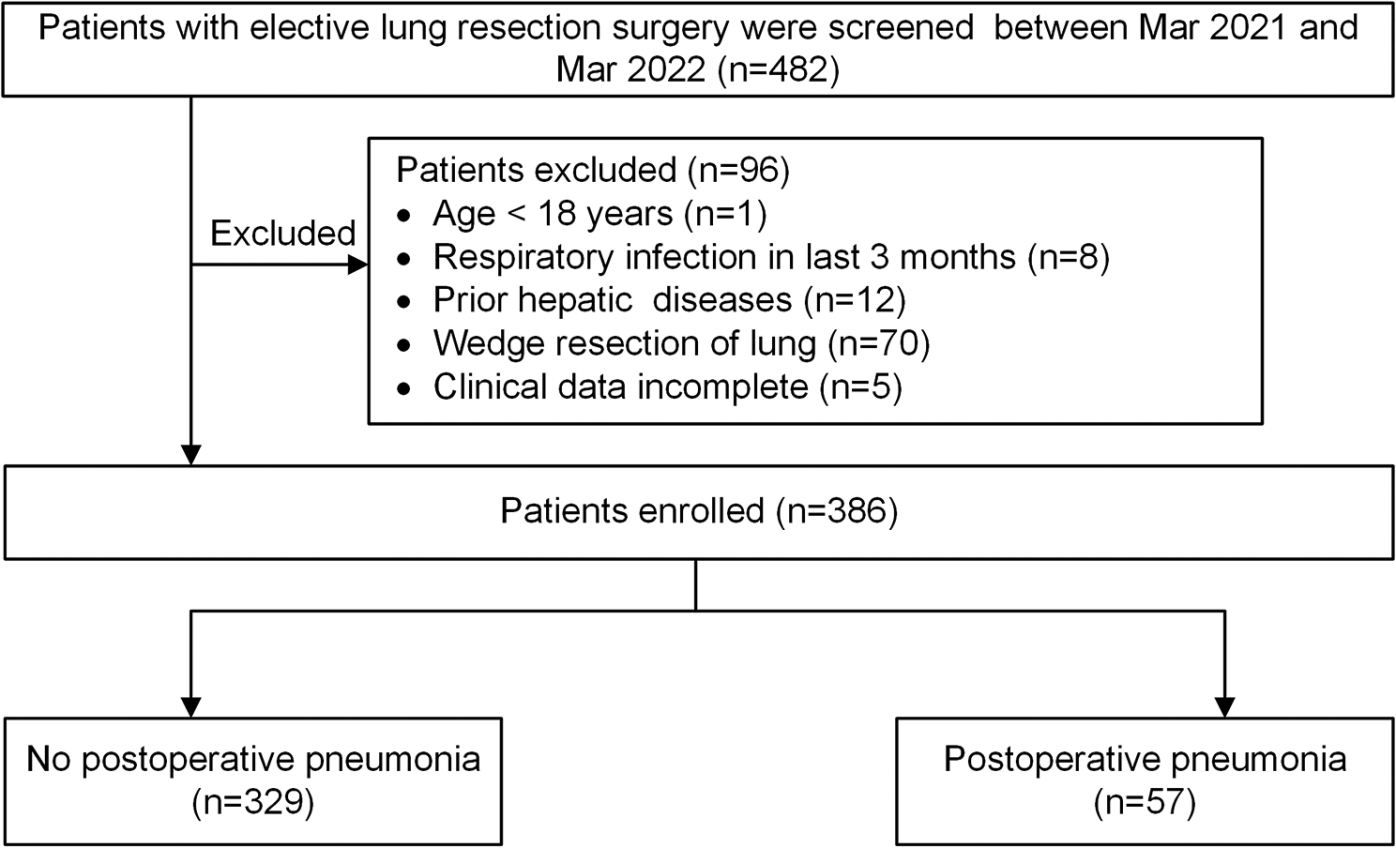
Flow chart

A total of 482 patients were screened for eligibility between March 2021 and March 2022. After applying the inclusion and exclusion criteria, 386 eligible patients were included (Fig. 1), with a median age of 62.0 years (IQR: 55.0, 68.0), of whom 156 (40.4%) were male. Among the patients, 59 (15.3%) patients experienced coronary heart disease and 49 (12.7%) experienced COPD; 80 (20.7%) underwent segmentectomy, 306 (79.3%) underwent lobectomy. Of the entire patient cohort, 57 (14.8%) patients presented with postoperative pneumonia within 7 days after surgery.

There were no significant differences in the baseline characteristics between postoperative pneumonia and no postoperative pneumonia groups in terms of age, gender, BMI, ASA physical status, smoking and alcohol status, hypertension, diabetes mellitus, COPD, type of surgery, duration of surgery, and so on. However, patients with postoperative pneumonia had a higher rate of coronary heart disease as a comorbidity, than did those without postoperative pneumonia (26.3% vs. 13.4%, *P* = 0.021) (Table 1). About preoperative laboratory findings, the median SII (×10^9^/L) and prealbumin (mg/L) values were 334.5 (IQR: 234.0, 461.0) and 253.5 (IQR: 230.0, 282.0), respectively. Compared to patients without postoperative pneumonia, those with postoperative pneumonia had higher preoperative SII scores (388.0 [IQR: 292.3, 468.3] vs. 307.0 [IQR: 226.0, 456.5], *P* = 0.012) (Table 1).

**Table 1:** Clinical characteristics of the patients by incident postoperative pneumonia

Variables	Overall (*n* = 386)	Postoperative pneumonia (*n* = 57)	No postoperative pneumonia (*n* = 329)	*P* value
Demographics				
Age, y	62.0 (55.0, 68.0)	62.0 (55.0, 68.0)	61.0 (56.0, 68.3)	0.536
Male (%)	156 (40.4)	26 (45.6)	130 (39.5)	0.471
BMI, kg/m^2^	24.0 (22.0, 26.2)	23.8 (22.3, 25.7)	24.0 (21.8, 26.2)	0.436
ASA physical status (%)				
Class I–II	308 (79.8)	45 (78.9)	263 (79.9)	0.995
Class III	78 (20.2)	12 (21.1)	66 (20.1)	
Smoking status (%)	47 (12.2)	9 (15.8)	38 (11.6)	0.494
Alcohol (%)	46 (11.9)	10 (17.5)	36 (10.9)	0.061
Comorbidity				
Hypertension (%)	169 (43.8)	29 (50.9)	140 (42.6)	0.052
Diabetes mellitus (%)	47 (12.2)	6 (10.5)	41 (12.5)	0.847
Coronary heart disease (%)	59 (15.3)	15 (26.3)	44 (13.4)	0.021
COPD (%)	49 (12.7)	10 (17.5)	39 (11.9)	0.329
Type of surgery				
Segmentectomy (%)	80 (20.7)	7 (12.3)	73 (22.2)	0.127
Lobectomy (%)	306 (79.3)	50 (87.7)	256 (77.8)	
Duration of surgery, min	120.0 (93.0, 160.0)	140.0 (100.0, 180.0)	120.0 (90.0, 155.3)	0.102
Blood loss, mL	50.0 (50.0, 59.0)	50.0 (30.0, 62.5)	50.0 (50.0, 59.3)	0.633
Total fluids; ml/kg/h	9.0 (7.0, 12.0)	8.0 (6.0, 11.0)	9.0 (7.0, 12.0)	0.094
Preoperative laboratory findings				
Prealbumin, mg/L	253.5 (230.0, 282.0)	252.0 (228.8, 280.3)	260.0 (232.0, 293.0)	0.055
Albumin, g/L	39.5 (37.5, 41.1)	40.0 (37.7, 41.6)	39.5 (37.4, 41.1)	0.233
Neutrophil, ×10^9^/L	2.9 (2.3, 3.7)	3.2 (2.5, 3.8)	2.9 (2.3, 3.6)	0.332
Lymphocyte, ×10^9^/L	1.7 (1.4, 2.0)	1.7 (1.4, 2.1)	1.7 (1.4, 2.0)	0.322
Platelet, ×10^9^/L	194.0 (161.0, 232.0)	205.0 (171.5, 247.0)	193.0 (159.0, 231.0)	0.198
Hemoglobin, g/L	132.0 (122.0, 141.0)	132.0 (125.8, 142.3)	133.0 (121.0, 141.0)	0.432
Total bilirubin, μmol/L	13.0 (10.2, 16.5)	14.0 (10.4, 17.5)	12.6 (10.2, 16.2)	0.221
Preoperative SII, ×10^9^/L	334.5 (234.0, 461.0)	388.0 (292.3, 468.3)	307.0 (226.0, 456.5)	0.012

Patient characteristics are expressed as n (%), mean ± standard deviation, or median (interquartile range)

*Abbreviations* BMI: body mass index; ASA: American Society of Anesthesiologists; COPD: chronic obstructive pulmonary disease; SII, systemic immune-inflammation index

### Association between SII or prealbumin and postoperative pneumonia

We next evaluated the clinical relationship between SII or prealbumin and postoperative pneumonia using univariate and multivariate logistic analyses. When investigating the predictive value of preoperative SII or prealbumin as continuous variable, the univariate analysis showed that age, gender, BMI, ASA physical status, smoking and alcohol status, hypertension, diabetes mellitus, COPD, type of surgery, duration of surgery, blood loss, and total fluids had no predictive significance. Moreover, coronary heart disease [odds ratio (OR): 2.31; 95% confidence interval (CI): 1.18–4.52; *P* = 0.014] and preoperative SII (OR: 1.43, 95% CI: 1.43 (1.22–3.37), *P* = 0.008) were positively correlated with the occurrence of postoperative pneumonia significantly, whereas preoperative prealbumin (OR: 0.86, 95% CI: 0.43–0.95, *P* = 0.017) was negatively correlated with postoperative pneumonia. Then, after adjustment of confounding factors, preoperative SII was associated with an increased risk of postoperative pneumonia (OR: 1.38, 95% CI: 1.19–2.83, *P* = 0.011). The prealbumin remained as an independent protective predictor of postoperative pneumonia in the adjusted analysis (OR: 0.80, 95% CI: 0.37–0.89, *P* = 0.023) (Table 2).

**Table 2:** Association between preoperative SII or prealbumin as continuous variable and postoperative pneumonia

Variables	Univariate analysis		Multivariate analysis	
	OR (95% CI)	*P* value	OR (95% CI)	*P* value
Age	1.01 (0.98–1.04)	0.691		
Sex (male vs. female)	1.28 (0.73–2.26)	0.387		
BMI	0.96 (0.88–1.06)	0.435		
ASA physical status (Class I–II vs. Class III)	1.06 (0.53–2.12)	0.863		
Smoking status (Yes vs. No)	1.44 (0.65–3.16)	0.368		
Alcohol (Yes vs. No)	1.73 (0.81–3.72)	0.160		
Hypertension (Yes vs. No)	1.40 (0.80–2.46)	0.244		
Diabetes mellitus (Yes vs. No)	0.83 (0.33–2.05)	0.680		
Coronary heart disease (Yes vs. No)	2.31 (1.18–4.52)	0.014	2.23 (1.22–4.82)	0.009
COPD (Yes vs. No)	1.58 (0.74–3.38)	0.237		
Type of surgery (Segmentectomy vs. Lobectomy)	0.49 (0.21–1.13)	0.094	0.59 (0.32–1.08)	0.121
Duration of surgery	1.00 (1.00–1.01)	0.106		
Blood loss	1.00 (1.00–1.00)	0.673		
Total fluids	0.94 (0.87–1.01)	0.087	0.92 (0.85–1.05)	0.132
Preoperative prealbumin	0.86 (0.43–0.95)	0.017	0.80 (0.37–0.89)	0.023
Preoperative SII	1.43 (1.22–3.37)	0.008	1.38 (1.19–2.83)	0.011

*Abbreviations* OR: odds ratio; CI: confidence interval; BMI: body mass index; ASA: American Society of Anesthesiologists; COPD: chronic obstructive pulmonary disease; SII, systemic immune-inflammation index

The predictive performance of SII or prealbumin as categorical variable was also been evaluated. Results from the ROC analysis showed that the optimal cut-off points for the SII and prealbumin were set at 261 and 227 for predicting postoperative pneumonia. Consequently, the SII and prealbumin were stratified into low and high groups for subsequent analyses (Table 3). The results of univariate and multivariate logistic analyses revealed that both preoperative SII and prealbumin were independent influencing factors for predicting postoperative pneumonia (Supplementary Table 1). Therefore, we proposed that both preoperative SII and prealbumin, whether they are labeled “continuous” or “categorical”, were related to the the occurrence of postoperative pneumonia.

**Table 3:** Results of ROC analysis of preoperative SII, prealbumin, and combined detection for predicting postoperative pneumonia

Predictors	Cut-off value	Youden index	Sensitivity	Specificity	AUC (95% CI)	*P* value
Prealbumin	227	0.14	65.2%	54.1%	0.60 (0.57–0.68)	0.001*
SII	261	0.20	78.3%	42.8%	0.69 (0.65–0.75)	0.037^†^
Combined detection	50	0.21	81.1%	78.6%	0.79 (0.71–0.86)	0.001^#^

*Abbreviations* ROC: receiver operating characteristic; SII, systemic immune-inflammation index; AUC: area under the ROC curve; CI: confidence interval * indicates Combined detection comparison with Prealbumin † indicates SII comparison with Prealbumin # indicates Combined detection comparison with SII

### Predictive development and validation by ROC and DCA

**Fig. 2:**
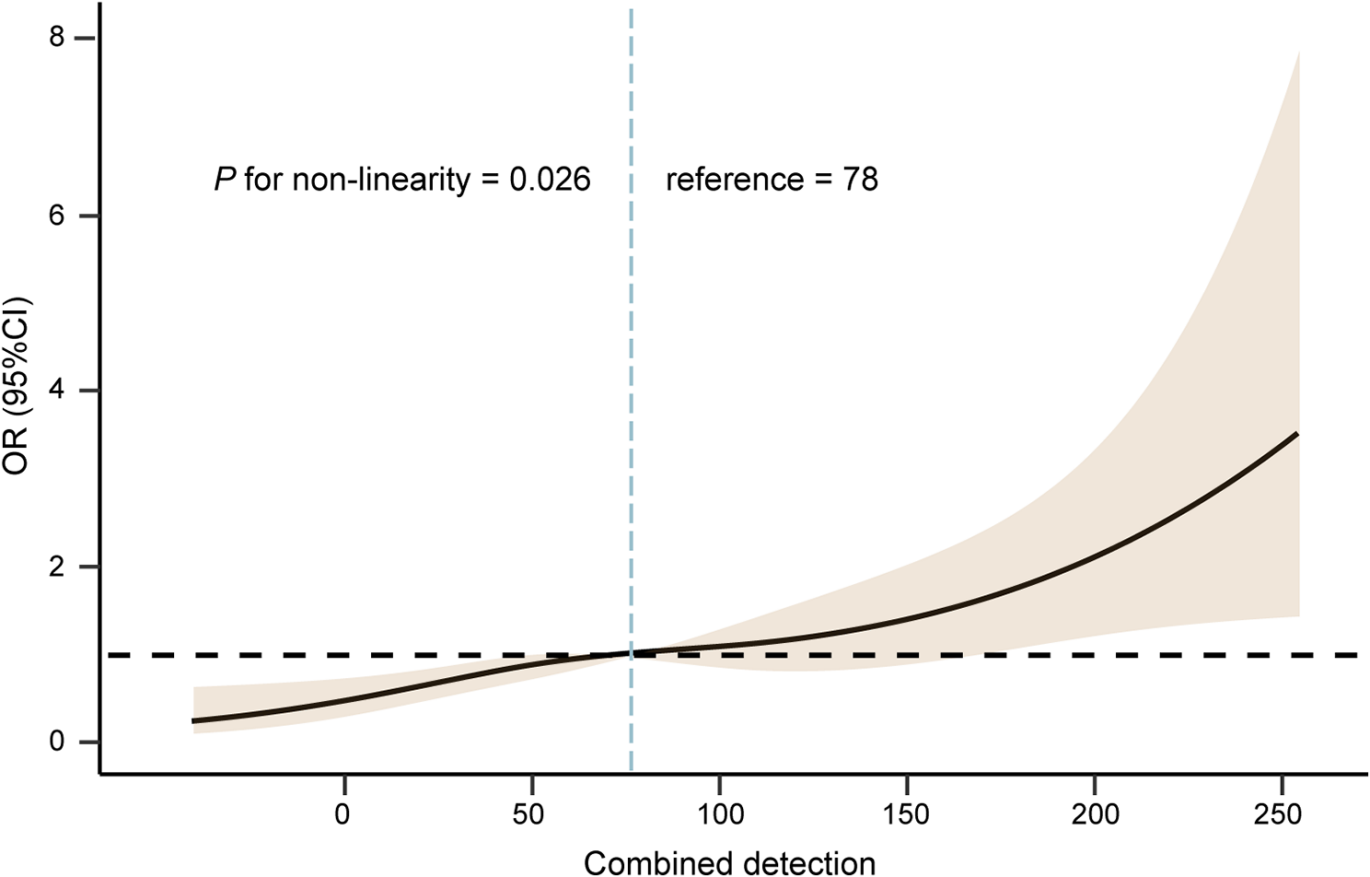
Multivariate adjusted odds ratio for postoperative pneumonia is based on restricted cubic spline analysis with three knots at the 25th, 75th percentiles, and the reference point at the median of combined detection of SII and prealbumin. Solid lines indicate point estimates on the relationship between combined detection and postoperative pneumonia, and the dashed lines represent 95% CI estimation. OR, odds ratio; SII, systemic immune-inflammation index

**Fig. 3:**
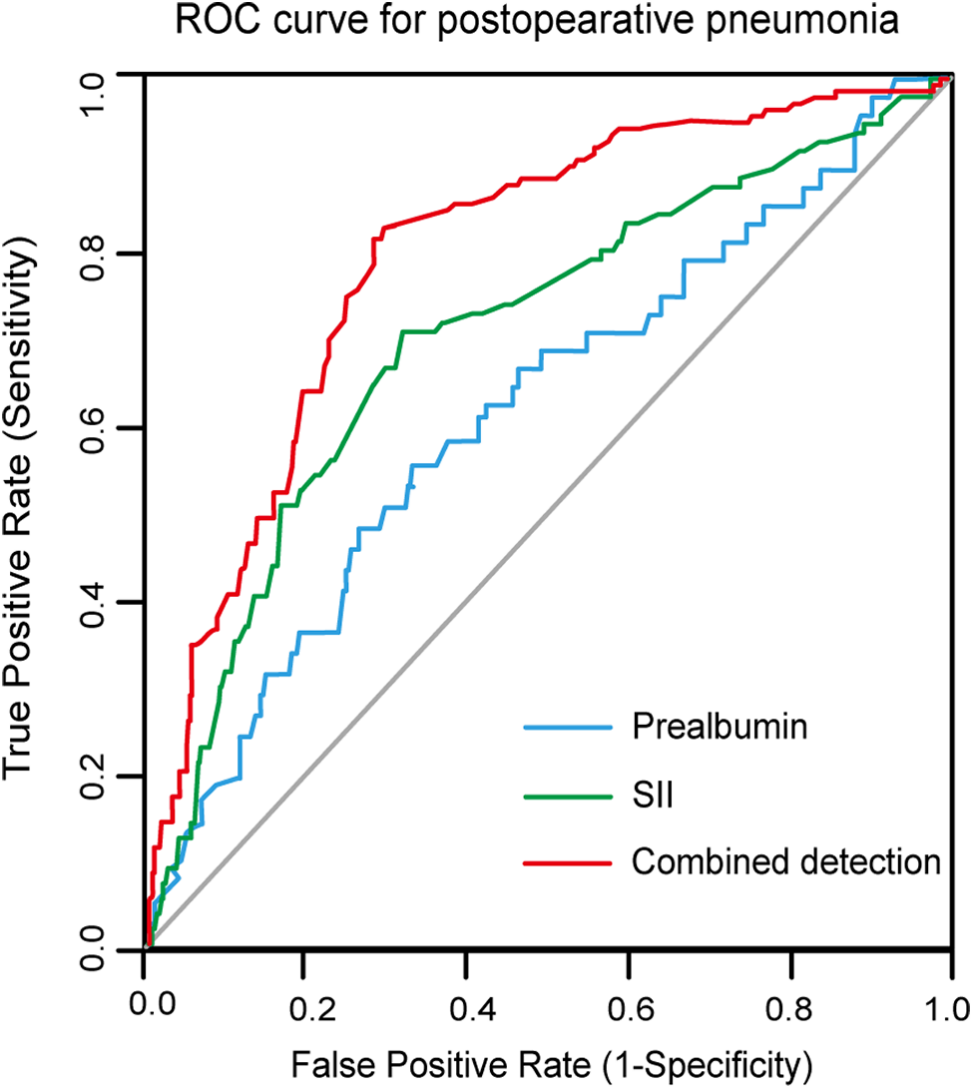
ROC curve for postoperative pneumonia. ROC, receiver operating characteristic curve; SII, systemic immune-inflammation index

**Fig. 4:**
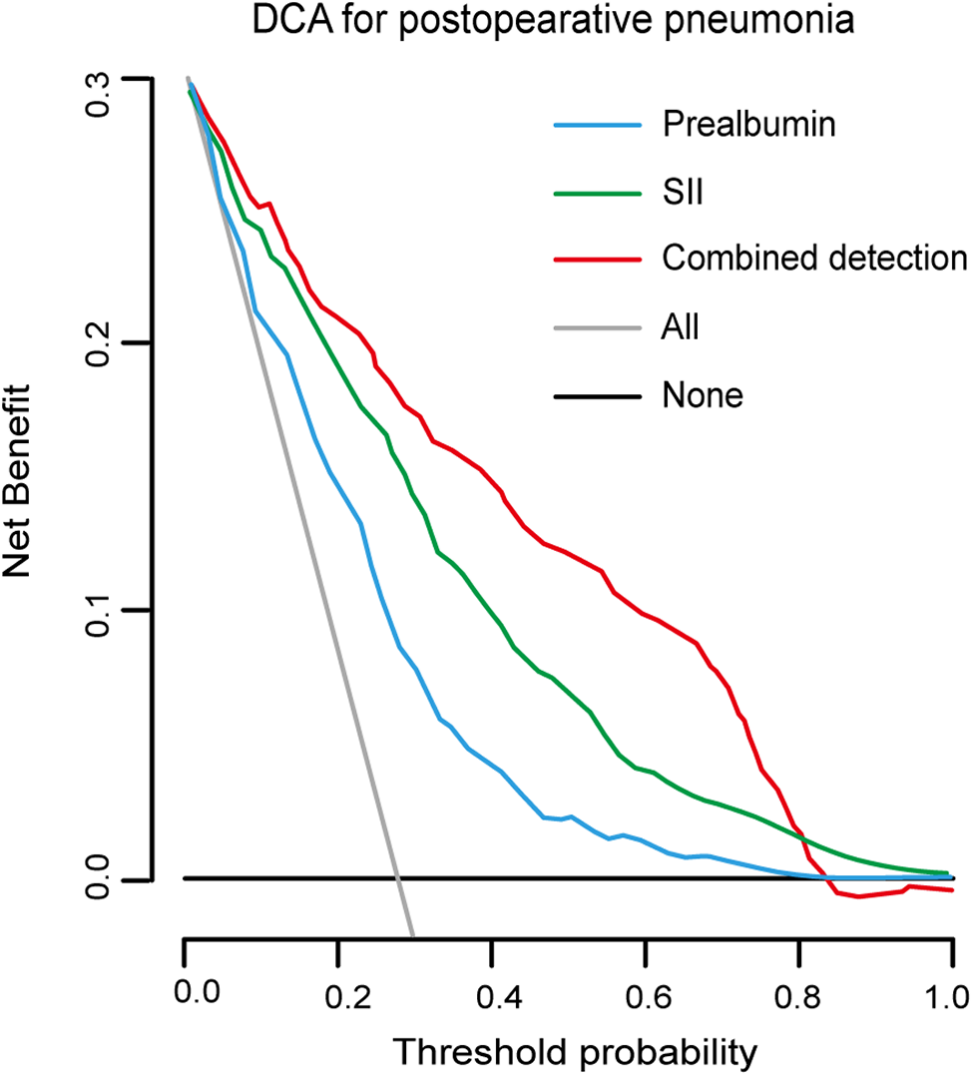
DCA for postoperative pneumonia. DCA, decision curve analysis; SII, systemic immune-inflammation index

The discrimination ability was the capability to identify patients with postoperative pneumonia and patients without that. The discrimination ability was measured by the AUC. Preoperative SII or prealbumin predicted postoperative pneumonia with AUC of 0.69 (95% CI: 0.65–0.75) and 0.60 (95% CI: 0.57–0.68), respectively (Table 3). Given that both preoperative SII and prealbumin were significantly independent predictive factors, we next included preoperative SII together with prealbumin as continuous variable in logistic regression analysis to fit a prediction equation for evaluating the predictive performance of integrating detection. The newly developed equation was established as follows: Y = 5.218 + 0.382 × SII – 0.236 × prealbumin (Supplementary Table 2). Restricted cubic spline analysis indicated an adjusted dose-dependent relationship between combined detection and the risk of genesis of postoperative pneumonia (*P* for non-linearity = 0.026; Fig. 2). Using the ROC analysis, the optimal cut-off point for the combined detection was 50, the Youden index was 0.21. Compared to SII or prealbumin, the combined detection showed the highest sensitivity (81.1%) and specificity (78.6%), with highest AUC of 0.79 (95% CI: 0.71–0.86, *P* < 0.05 for all) (Table 3; Fig. 3). Additionally, we conducted the DCA to evaluate the clinical utility and benefit. As illustrated in Fig. 4, the decision curve of the combined detection showed a positive net benefit over the none and all lines for predicted probability thresholds between 0 and 0.8, as well as over preoperative SII or prealbumin alone, indicating better clinical practicability for predicting postoperative pneumonia of surgical lung resection patients.

## Discussion

Using this cohort of patients scheduled for lung resection surgery, we evaluated the predictive value of the inflammatory biomarker SII combined with the nutritional biomarker prealbumin for the occurrence of postoperative pneumonia. Our results revealed that both elevated preoperative SII and decreased prealbumin were independent risk factors for postoperative pneumonia. More importantly, the prediction of postoperative pneumonia from SII combined with prealbumin offered better performance compared to either biomarker alone.

In this study, we reported the incidence of postoperative pneumonia among patients after lung resection surgery to be 14.8%. The incidence of postoperative pneumonia was aligned with previously reported rates of 2–18% in patients undergoing lung resection surgery [14–20]. At present, whereas the pathogenesis of postoperative pneumonia has not yet been elucidated, perioperative inflammation and nutritional status are potentially critical determinants of pneumonia susceptibility [21, 22].

SII has been developed based on a combination of neutrophils, lymphocytes, and platelets, which reflects the systemic inflammatory response and oxidative stress status [23, 24]. High SII is correlated with worse survival outcomes among patients with lung-invasive mucinous adenocarcinoma [25]. Alternatively, a retrospective study in 2023 found that the preoperative SII was effective in predicting the occurrence of postoperative lung infections [7] in patients undergoing lung cancer surgery. In contrast to 2023 study above, our study showed that preoperative SII indicated a moderate prediction capability for postoperative pneumonia (AUC = 0.69) in patients with lung resection surgery. Potential reasons for this discrepancy include differences in population characteristics, surgical procedures, and perioperative management. Our study differed from the 2023 study above in having longer operative time, and more complex surgical procedures (without pulmonary wedge resection). These adverse factors might cause broader pathophysiological alterations, thereby leading to increased difficulty in the prediction of postoperative pneumonia. Previous studies indicated that co-detecting multiple biomarkers, such as CRP to prealbumin [11], CRP to albumin [26], and modified Glasgow prognostic score (mGPS) [27–29], could refine and ultimately improve the prediction accuracy of postoperative complications or survival outcomes. Our results, consistent with others [10, 11], revealed that falling preoperative prealbumin was an independent predictor of postoperative pneumonia, although it only displayed limited prediction power (AUC = 0.60). Therefore, we proposed SII combined with prealbumin that could proportionately integrate inflammation and malnutrition effects to improve predictive capability further.

Our results demonstrated that combined detection of SII and prealbumin was superior to both SII and prealbumin in predicting postoperative pneumonia among patients undergoing lung resection surgery (AUC = 0.79). Moreover, according to the DCA, the combined detection had broader clinical applicability and more clinical benefit than preoperative SII or prealbumin alone. The notable advantage of this combined detection in our study was the proposition that the addition of prealbumin to SII bestowed a simultaneous assessment of nutritional status besides inflammatory pathway. The tight linkage between inflammation and malnutrition may account for the improvement of prediction capability and clinical benefit [30]. Inflammatory signals could inhibit the synthesis of some acute-phase reactant proteins, such as prealbumin [31, 32]. In addition, falling serum prealbumin suggested that the patients were in a state of underlying malnutrition, which could significantly promote the release of systemic proinflammatory mediators, and interfere with immune cell metabolism, thereby resulting in immunological cascades, infection, or multiple organ damages [33–35]. As seen, the combined detection of SII and prealbumin might amplify the inflammation and malnutrition effects, thus exacerbating postoperative pulmonary infections. Comprehensive evaluation of inflammatory and nutritional characteristic changes was a necessary and promising strategy for risk stratification in surgical lung resection patients.

There are several inevitable limitations in this study. Firstly, it is a retrospective study, and therefore, we could only observe the association between SII or prealbumin and postoperative pneumonia without causality. Secondly, this study was conducted at a single center with a limited sample size, potentially introducing selection bias and restricting the generalizability of the conclusions. Thirdly, despite efforts to adjust for the potential confounding effects, unknown or unmeasured confounders may affect our results to some extent. Fourthly, CRP to prealbumin, CRP to albumin, and mGPS are also combined indicators, which reflect inflammatory and nutritional status. However, C-reactive protein was not a routine blood examination in clinical practice, these data were not available in our study. Additional studies are warranted to assess the differences in the predictive capacity of CRP to prealbumin, CRP to albumin, mGPS, and SII combined with prealbumin. Fifthly, the predictive value of this novel combined detection needs to be prospectively validated in different external cohorts. Finally, more research is needed to further determine which biomarker or different combination of biomarkers can achieve the optimal performance in predicting postoperative pneumonia.

## Conclusion

Combined detection of preoperative SII and prealbumin is a novel integration of biomarkers available from routine blood examinations and reflects inflammation and malnutrition effects. Combined detection may significantly improve prediction capability for postoperative pneumonia, indicating broad clinical applicability in risk stratification of postoperative pneumonia in surgical lung resection patients.

### Electronic supplementary material

Below is the link to the electronic supplementary material.

Supplementary Material 1

## Data Availability

The dataset used and/or analyzed during the present study are available from the corresponding author on reasonable request.

## Abbreviations

SII: Systemic immune-inflammation index
POP: Postoperative pneumonia
ROC: Receiver operating characteristic curve
DCA: Decision curve analysis
IQR: Interquartile range
OR: Odds ratio
CI: Confidence interval
CRP: C-reactive protein
STROBE: STrengthening the Reporting of OBservational studies in Epidemiology
BMI: Body mass index
ASA: American society of anesthesiologists
COPD: Chronic obstructive pulmonary disease
AUC: Area under the ROC curve
mGPS: modified Glasgow prognostic score
