# Supplementary material for: Predictive significance of systemic immune-inflammation index combined with prealbumin for postoperative pneumonia following lung resection surgery
Source: BMC Pulm Med. 2024 Jun 11;24:277. doi: 10.1186/s12890-024-03086-7 (PMC11167804; doi:10.1186/s12890-024-03086-7)
Supplement: Supplementary file 1 — Supplementary Material 1 [file 12890_2024_3086_MOESM1_ESM.docx]

**SUPPLEMENTAL MATERIAL**

**Supplementary Table 1** Association between preoperative SII or prealbumin as categorical variable and postoperative pneumonia.

| Variables | Univariate analysis | | Multivariate analysis | |
| --- | --- | --- | --- | --- |
|  | OR (95% CI) | *P* value | OR (95% CI) | *P* value |
| Age | 1.01 (0.98–1.04) | 0.691 |  |  |
| Sex (male vs female) | 1.28 (0.73–2.26) | 0.387 |  |  |
| BMI | 0.96 (0.88–1.06) | 0.435 |  |  |
| ASA physical status (Class I–II vs Class III) | 1.06 (0.53–2.12) | 0.863 |  |  |
| Smoking status (Yes vs No) | 1.44 (0.65–3.16) | 0.368 |  |  |
| Alcohol (Yes vs No) | 1.73 (0.81–3.72) | 0.160 |  |  |
| Hypertension (Yes vs No) | 1.40 (0.80–2.46) | 0.244 |  |  |
| Diabetes mellitus (Yes vs No) | 0.83 (0.33–2.05) | 0.680 |  |  |
| Coronary heart disease (Yes vs No) | 2.31 (1.18–4.52) | 0.014 | 2.56 (1.27–5.15) | 0.008 |
| COPD (Yes vs No) | 1.58 (0.74–3.38) | 0.237 |  |  |
| Type of surgery (Segmentectomy vs Lobectomy) | 0.49 (0.21–1.13) | 0.094 | 0.47 (0.20–1.10) | 0.083 |
| Duration of surgery | 1.00 (1.00–1.01) | 0.106 |  |  |
| Blood loss | 1.00 (1.00–1.00) | 0.673 |  |  |
| Total fluids | 0.94 (0.87–1.01) | 0.087 | 0.94 (0.87–1.01) | 0.073 |
| Preoperative prealbumin (< 227 vs ≥ 227) | 0.67 (0.33–0.89) | 0.021 | 0.73 (0.42–0.91) | 0.018 |
| Preoperative SII (< 261 vs ≥ 261) | 3.12 (1.43–6.81) | 0.004 | 3.35 (1.52–7.42) | 0.003 |
| Abbreviations: OR: odds ratio; CI: confidence interval; BMI: body mass index; ASA: American Society of Anesthesiologists; COPD: chronic obstructive pulmonary disease; SII, systemic immune-inflammation index. | | | | |

**Supplementary Table 2** Results of the multivariate analysis of preoperative SII together with prealbumin as continuous variable associated with postoperative pneumonia.

| Variables | *B* | OR (95% CI) | *P* value |
| --- | --- | --- | --- |
| Preoperative prealbumin | –0.236 | 0.79 (0.43–0.91) | 0.021 |
| Preoperative SII | 0.382 | 1.47 (1.24–3.02) | 0.009 |
| Abbreviations: OR: odds ratio; CI: confidence interval; SII, systemic immune-inflammation index. | | | |
